# Normative prospective data on automatically quantified retinal morphology correlated to retinal function in healthy ageing eyes by two microperimetry devices

**DOI:** 10.1111/aos.17434

**Published:** 2024-12-27

**Authors:** Klaudia Birner, Leonard M. Coulibaly, Johannes Schrittwieser, Irene Steiner, Hamza Mohamed, Simon Schürer‐Waldheim, Markus Gumpinger, Hrvoje Bogunovic, Ursula Schmidt‐Erfurth, Gregor S. Reiter

**Affiliations:** ^1^ Department of Ophthalmology and Optometry Medical University of Vienna Vienna Austria; ^2^ Center for Medical Data Science, Institute of Medical Statistics Medical University of Vienna Vienna Austria; ^3^ Aritificial Intelligence Institute, Center for Medical Data Science Medical University of Vienna Vienna Austria

**Keywords:** artificial intelligence, deep‐learning, ellipsoid zone, Microperimetry, optical coherence tomography

## Abstract

**Purpose:**

The relationship between retinal morphology, as assessed by optical coherence tomography (OCT), and retinal function in microperimetry (MP) has not been well studied, despite its increasing importance as an essential functional endpoint for clinical trials and emerging therapies in retinal diseases. Normative databases of healthy ageing eyes are largely missing from literature.

**Methods:**

Healthy subjects above 50 years were examined using two MP devices, MP‐3 (NIDEK) and MAIA (iCare). An identical grid, encompassing 45 stimuli was used for retinal sensitivity (RS) assessment. Deep‐learning‐based algorithms performed automated segmentation of ellipsoid zone (EZ), outer nuclear layer (ONL), ganglion cell layer (GCL) and retinal nerve fibre layer (RNFL) from OCT volumes (Spectralis, Heidelberg). Pointwise co‐registration between each MP stimulus and corresponding location on OCT was performed via registration algorithm. Effect of age, eccentricity and layer thickness on RS was assessed by mixed effect models.

**Results:**

Three thousand six hundred stimuli from twenty eyes of twenty patients were included. Mean patient age was 68.9 ± 10.9 years. Mean RS for the first exam was 28.65 ± 2.49 dB and 25.5 ± 2.81 dB for MP‐3 and MAIA, respectively. Increased EZ thickness, ONL thickness and GCL thickness were significantly correlated with increased RS (all *p* < 0.001). Univariate models showed lower RS with advanced age and higher eccentricity (both *p* < 0.05).

**Conclusion:**

This work provides reference values for healthy age‐related EZ and ONL‐thicknesses without impairment of visual function and evidence for RS decrease with eccentricity and increasing age. This information is crucial for interpretation of future clinical trials in disease.

## 1 INTRODUCTION

During the last decades, constant technological advancements have revolutionized the use of Optical Coherence tomography (OCT) in clinical ophthalmology (Fujimoto & Swanson, 2016; Huang et al., 1991). The 3‐dimensional, high‐resolution depiction of precise retinal morphology offered by OCT imaging has become an indispensable tool for the diagnosis and the monitoring of patients with macular disease (Sadda et al., 2018; Spaide et al., 2020). With the introduction of artificial intelligence‐based algorithms a quantitative assessment of disease‐specific structural changes on OCT becomes accessible in a comprehensive and time‐saving manner (Reiter et al., 2024; Schmidt‐Erfurth & Waldstein, 2016). Meanwhile, the most common endpoint in clinical trials on retinal disease recognized by the European Medicines Agency (EMA) and the Federal Drug Agency (FDA) remains conventional best‐corrected visual acuity (BCVA) (Csaky et al., 2018). Even if widely accepted as gold standard, BCVA might be considered as an imprecise indicator for overall retinal functionality as it mostly mirrors visual impairment within the central macular millimetre (Csaky et al., 2018). This limitation becomes apparent in the assessment of retinal pathologies with a subclinical manifestation in the early disease stages due to small and/or peripheral development such as geographic atrophy or inherited retinal disease (K. Csaky et al., 2018).

Therefore, regulatory agencies have highlighted the need for novel clinical functional endpoints to address the shortfalls of BCVA regarding the setup of future clinical trials (Csaky et al., 2024).

Microperimetry (MP) offers a non‐invasive psychophysical assessment of pointwise retinal sensitivity (PWS). The incorporated eye‐tracking sensor and fundus camera allow the device to perform a comprehensive mapping of light sensitivity over the complete macular area (Pfau et al., 2021). Its usefulness in the monitoring of progressive neurodegenerative non‐foveal retinal modifications has been highlighted for numerous diseases, including age‐related macular degeneration (AMD) (Meleth et al., 2011) and inherited retinal disease like Stargardt disease(Schönbach et al., 2017) or retinitis pigmentosa (Iftikhar et al., 2018). Retinal sensitivity assessment by MP has therefore been discussed as a valuable additional functional clinical endpoint for clinical trials in above‐mentioned pathologies (Csaky et al., 2018; Iftikhar et al., 2018; Pfau et al., 2024; Schönbach et al., 2017).

Understanding the relation between topographic structural changes of retinal morphology and loss of visual function is a crucial step for the identification of disease promoting mechanisms. Further it enables a better evaluation of potential therapy benefits in clinical trials. Structure–function correlations including MP and high‐resolution OCT imaging have been performed for patients with macular disease (Montesano et al., 2017; Pfau et al., 2020; Saßmannshausen et al., 2018). Nonetheless, comprehensive reference values for precise structure–function correlation between layer morphology and MP for healthy subjects are limited, even though they constitute the basis of all potential pathologic developments.

The aim of this study is to determine reference values for PWS and correlated structural morphology in healthy subjects using the most common commercially available MP devices and high‐resolution OCT imaging. These references may prove to be valuable for the interpretation of previous and future clinical trials where MP will be used as additional endpoint.

## 2 METHODS

This study was performed in accordance with the tenets of the declaration of Helsinki and was approved by the institutional ethics committee of the Medical University of Vienna.

### 2.1 Study population

Eyes of subjects 50 years of age and older with no diagnosed retinal disease were included in the analysis. Recruitment was based on accompanying age‐matched subjects of macula outpatient clinic patients and/or healthy fellow eyes of unilateral ocular pathologies. Consequently, subjects with a previous history of anti‐VEGF therapy, neurodegenerative altercations of any retinal layer, signs of beginning glaucoma or any other structural altercation with a possible relation to disease activity were excluded from participating in the study. Further a diagnosis of retinal vein occlusion (RVO) in the fellow eye was considered as exclusion criteria. Presence of isolated drusen smaller than 63 μm, previously called drupelets in the Beckman classification, was considered a physiologic sign of ageing and was tolerated within the study population (Ferris et al., 2013). One eye per patient was included. When both eyes were eligible, the eye with better OCT imaging quality was chosen.

### 2.2 Imaging protocol

**FIGURE 1:**
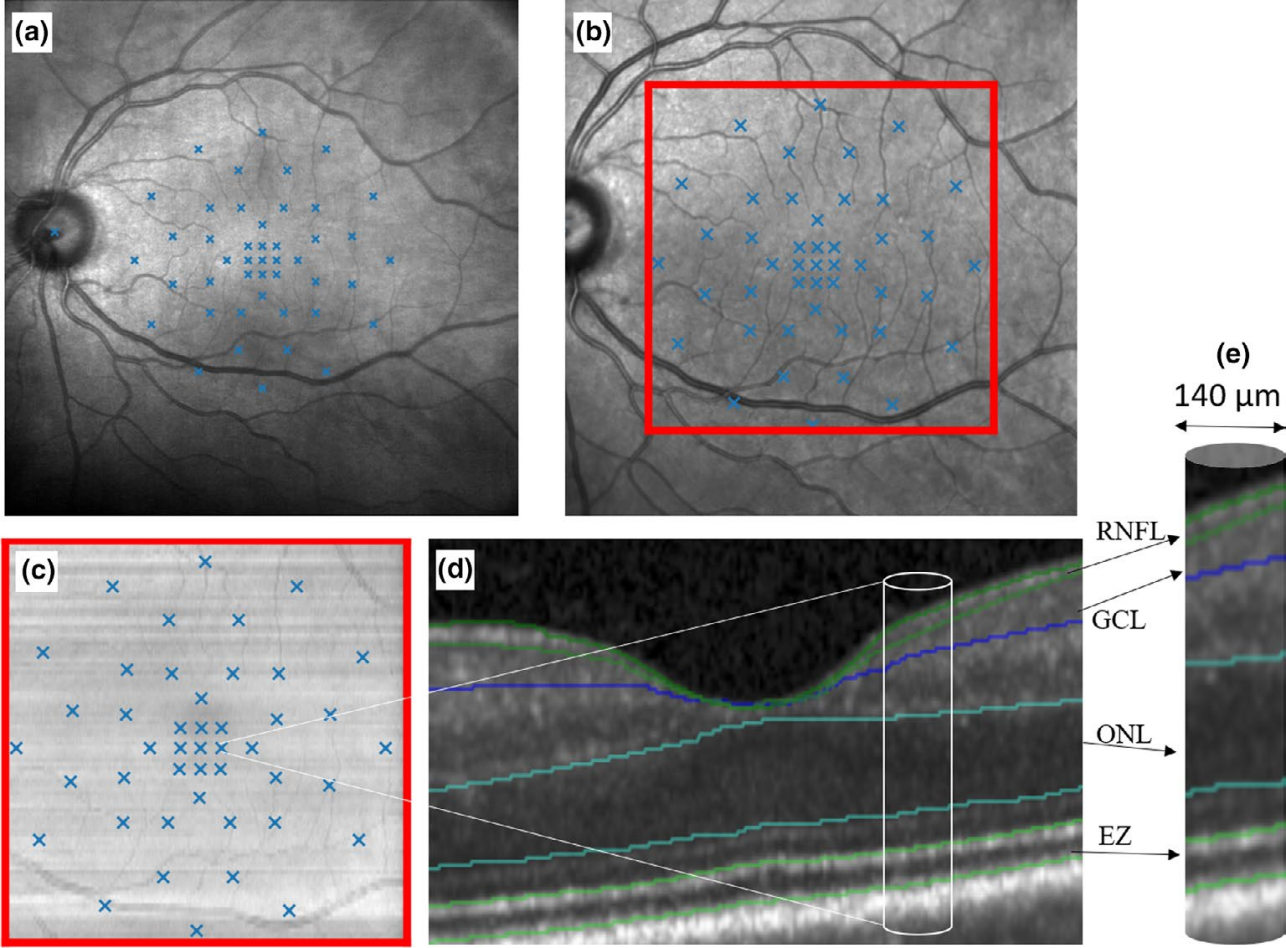
The following schematic example illustrates the process of stimulus co‐registration using the MAIA device. (a) depicts the near‐infrared reflectance (NIR) of the MAIA with the 45 projected stimuli. (b) depicts the NIR of the Spectralis OCT obtained at each examination. On this NIR the region of interest, encompassing 20×20°, is delineated by the red box. The overlay of the NIR (MAIA) to the NIR (OCT) is performed by an algorithm that registers retinal vessels using a deep‐learning algorithm (b). As the NIR obtained with the Spectralis is already aligned with the OCT by the Heidelberg software, no further alignment is necessary, and the grid is projected onto the en‐face OCT (c). The result is a stimulated section at B‐scan level with a radius of 70 μm and a diameter of 140 μm, as illustrated in (d) and (e). This approach allows for the direct correlation of sensitivity values with the structural characteristics, for example, layer thickness of the stimulated region.

Eligibility was ensured via imaging on Spectral‐domain OCT (SD‐OCT) volume scan using a Heidelberg Spectralis HRA + OCT (Heidelberg Engineering, Heidelberg, Germany) with a 20×20° macular cube, consisting of 97 high‐resolution B‐scans (1024 Ascan) with an automatic real time (ART) value of 16 frames centred on the fovea. At the same visit two successive MP examinations were performed on the MP‐3 (NIDEK CO., Ltd., Gamagori, Japan) and MAIA (MAIA, iCare S.p.A., Padova, Italy) resulting in a total of four examinations. The order of device acquisition was randomized, and a 10‐minute break was held between each examination. A grid consisting of 45 Goldmann III stimuli with a 4–2 staircase strategy was used for all four MP examinations per patient as displayed in Figure 1. The standard testing mode was selected for each with MAIA in mesopic testing conditions (background 4 asb 1.27 cd/m^2^) and MP‐3 in photopic testing conditions (background 31.4 asb, 10 cd/m^2^). Our in‐house fovea‐centred grid had nine stimuli within the foveal region, 12 stimuli in the parafoveal region and 20 stimuli were in the perifoveal EDTRS region with the remaining four stimuli on the border between the parafoveal and perifoveal region (Coulibaly et al., 2024). An example of the in‐house developed stimulation pattern on both devices was previously published by Coulibaly et al. (Coulibaly et al., 2024).

### 2.3 AI‐supported biomarker quantification

**FIGURE 2:**
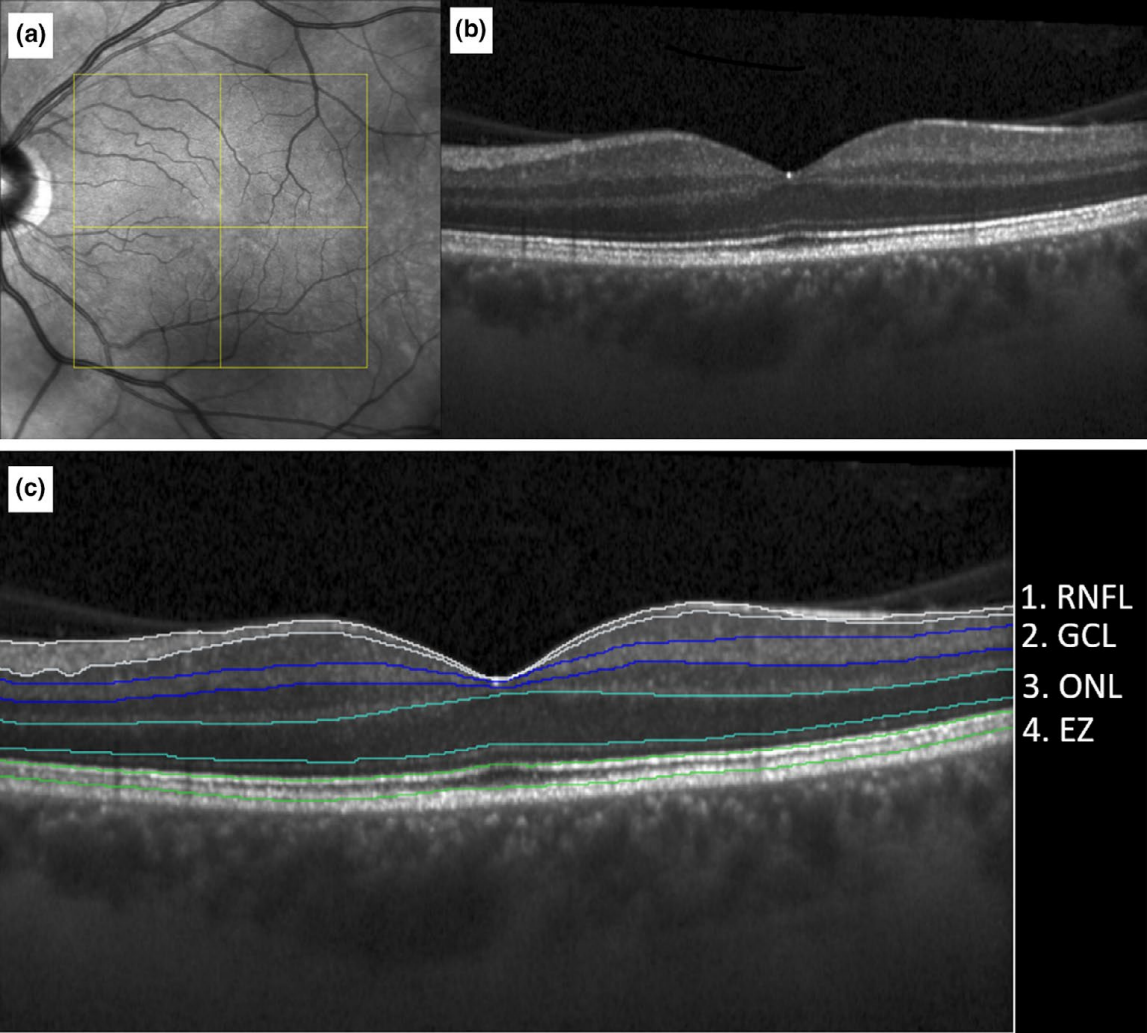
(a, b) Show the en‐face image and the B‐scan of a left eye of a healthy aged individual. (c) Shows the process of semi‐automated layer segmentation at the corresponding B‐scan level The layers were segmented using an in‐house layer segmentation algorithm followed by careful review by an expert reader. EZ, Ellipsoid zone; GCL, Ganglion cell layer; ONL, Outer nuclear layer; RNFL, Retinal nerve fibre layer.

Figure 2 displays an example of the layer segmentation on SD‐OCT. The ellipsoid zone (EZ), defined as the distance from the inner boundary of the EZ to the outer boundary of the interdigitation zone, was calculated fully automatically using a previously validated algorithm (Orlando et al., 2020). Thicknesses of the retinal nerve fibre layer (RNFL), the ganglion cell layer (GCL) and the outer nuclear layer (ONL) were automatically quantified in the whole volumetric OCT using an in‐house developed automated deep‐learning algorithm. Segmentation was carefully reviewed and regions with potential segmentation error due to vessels or visible Henle fibre layer (HFL) were manually corrected when indicated. The RNFL was defined as the distance between the internal limiting membrane and the inner boundary of the GCL. The GCL was defined as the distance between the outer boundary of the RNFL and the inner boundary of the inner plexiform layer. ONL was measured to the outer boundary of the outer plexiform to the inner boundary of the external limiting membrane (ELM) layer as previously proposed (Li et al., 2019). The HFL was included in the ONL thickness measurement (Sadigh et al., 2013).

### 2.4 Co‐registration between OCT and microperimetry

Registration between both MP devices and the respective OCT volume was done with an in‐house developed program as previously described (Birner et al., 2024). After exporting the data from each device, the pixel positions of each stimulus point on the MAIA near‐infrared reflectance (NIR) and MP3 colour fundus photography (CFP) were determined. Both the MP‐3‐CFP and the MAIA‐NIR were co‐registered with the NIR image acquired during the OCT B‐scan acquisition. The registration of the positions to the NIR is based on a U‐net that performs a vessel‐segmentation in MP images (both MP‐3 and MAIA) to define and connect the junctional points (Arikan et al., 2019). Detection of junctional points is performed using a MASK R‐CNN based network architecture that can find branches, bifurcations and crossings of vessels. Depending on the quality of the scan, at least three junctions between the MAIA‐NIR and the Spectralis NIR are required to correctly align the images. Then, the automated registration is carefully reviewed by an expert, who assures that the exact pixel positions correspond on both images. In case of misalignment of the respective MP stimuli points between the MAIA‐NIR/MP3‐CFP and the NIR, the junctional points are redefined by manual correction by marking the junctional points on both respective images and calculating the transformation matrix based on the “least square” error method. As the positions on the NIR obtained with the Spectralis device are already aligned with each corresponding OCT B‐scans as provided by the Heidelberg software, no further alignment is necessary, and the grid is projected onto the en‐face OCT. The result is the exact position of the MP stimulus at B‐scan level. For biomarker quantification at each respective MP stimulus position, OCT pixel spacing is used to determine all pixels surrounding each respective stimulus point within 70 μm radius (140 μm diameter) to quantify any biomarker, for example, layer thickness at the location of each of the 45 stimuli.

### 2.5 Statistical analysis

The primary endpoint of these analyses is the sensitivity estimates in decibel (dB). In a first step, univariate mixed models with patient as random factor were calculated (function lme, R‐package nlme_3.1–163) (Pinheiro, 2024). The independent variables were device and run as dichotomous variables and retinal eccentricity (R°), age, EZ thickness, ONL thickness, GCL thickness, RNFL‐thickness as metric variables. In a second step, variable selection was applied using the R‐function dredge (R‐package MUMIn_1.47.5) (Barton, 2023). The global model was a mixed model fitted by maximum likelihood (ML), with sensitivity as dependent variable and patient as random factor. The independent variables were those mentioned above. Additionally, interactions between EZ thickness and R°, ONL thickness and R°, age and EZ thickness as well as EZ thickness and ONL thickness were analysed in the univariate model.

For model selection, the Bayesian Information Criterion (BIC) was used. Due to missing values (outside the OCT's 20×20°), 3565 observations out of 3600 observations were included in the model. The final model was selected as the model with the lowest BIC. The estimates with 95% confidence limits and the p‐values of the final model (fitted by REML‐estimation) are reported. The correlations between independent variables were descriptively analysed by calculating spearman correlation coefficients across all stimuli (*n* = 3600 for the correlation between R° and Age and *n* = 3565 for the correlations with layer thickness).

Statistical analyses were carried out with R 4.3.2(R Core Team, 2023) and SPSS statistical package version 29 (SPSS Inc., Chicago, IL). The significance level has been set to alpha = 0.05. Note that the analyses are exploratory and hence the interpretation of the *p*‐values is descriptive.

## 3 RESULTS

The analysis collected 3600 MP stimulus points from 20 eyes of 20 subjects with a mean age of 68.9 years (SD ± 10.9 years). Table 1 presents the descriptive statistics of EZ thickness, ONL thickness, GCL thickness and RNFL thickness at the corresponding MP stimulus points for each run and each device. The mean PWS in the first run was 28.65 ± 2.49 dB and 25.5 ± 2.81 dB in the MP‐3 and MAIA, respectively.

**TABLE 1:** Descriptive statistics of normally distributed data with mean and standard deviation.

	MP‐3 run 1	MP‐3 run 2	MAIA run 1	MAIA run 2	Observations
Sensitivity (dB)	28.65 ± 2.49	29.1 ± 2.45	25.5 ± 2.81	25.38 ± 2.82	3.600
EZ thickness (μm)	33.54 ± 4.59	33.57 ± 4.58	33.30 ± 4.44	33.35 ± 4.44	3.565
ONL thickness (μm)	65.44 ± 19.79	65.49 ± 19.78	63.58 ± 19.38	63.72 ± 19.36	3.565
GCL thickness (μm)	33.22 ± 15.42	33.19 ± 15.42	33.25 ± 15.37	33.15 ± 15.38	3.565
RNFL thickness (μm)	27.90 ± 16.16	27.91 ± 16.13	28.81 ± 17.63	28.90 ± 17.68	3.565

Abbreviations: EZ, photoreceptor; GCL, ganglion cell layer; ONL, outer nuclear layer; RNFL, retinal nerve fibre layer.

Table 2 shows the descriptive statistics for each biomarker at the corresponding MP stimulus points in the foveal, parafoveal and perifoveal region for both MP devices and descriptive statistics of each biomarker over the entire OCT volume in the foveal, parafoveal and perifoveal regions.

**TABLE 2:** Descriptive statistics by location of microperimetry (MP) stimulus points in the foveal centre, parafovea and perifovea, and by total volume.

		Pointwise			Overall	
Macular region (μm)	Fovea	Parafovea	Perifovea	Fovea	Parafovea	Perifovea
EZ thickness (μm)	35.60 ± 5.30	34.30 ± 4.41	32.02 ± 3.62	35.48 ± 4.42	34.32 ± 3.60	31.94 ± 2.63
ONL thickness (μm)	93.43 ± 14.57	68.86 ± 10.69	49.94 ± 8.88	95.18 ± 9.67	67.93 ± 6.18	50.22 ± 5.36
GCL thickness (μm)	22.05 ± 14.19	48.93 ± 10.90	27.05 ± 9.53	20.86 ± 5.20	48.22 ± 7.20	27.01 ± 4.10
RNFL thickness (μm)	11.45 ± 4.96	23.21 ± 7.30	38.87 ± 18.22	11.40 ± 2.46	24.00 ± 2.98	38.78 ± 5.58
*n*	720	960	1565	20	20	20

*Note*: For the pointwise layer thicknesses, four points located on the border of the respective areas were not included.

### 3.1 Pointwise structure–function correlation

Table 3 demonstrates the results from the univariate and multivariable mixed effect models. When tested with the MAIA device patients demonstrated significantly lower PWS compared to MP‐3 (−3.335 [CI: −3.48; −3.19] dB). EZ thickness (0.05 dB/ μm [CI: 0.025–0.037]), ONL thickness (0.03 dB/ μm [0.025–0.037]) and GCL thickness (0.028 dB/ μm [0.022–0.033], all *p* < 0.0001) were all significantly associated with increased PWS see Table 3 and Figure S1. Interestingly, the thickness of the macular RNFL showed a negative, but clinically not relevant impact on foveal sensitivity (−0.01 dB/μm *p* = 0.0004) with expected physiological changes in RNFL thickness across the nasal, temporal superior and inferior regions of the macular cube (Figure S2). Both, the multivariable and the univariate model, showed consistent estimates for each respective layer thickness (all *p* < 0.0001). The univariate models demonstrated reduced PWS with increasing eccentricity and age whereby a one‐degree increase in eccentricity was associated with a—0.276 dB decrease in PWS (*p* < 0.0001). For this study, a 20×20° frame of the OCT was used, where the outer edge of the scan at 10° estimated PWS measurements of −2.76 dB (Table S1 and Figure S3). Age reduced PWS with an estimate of −0.053 dB/year (*p* = 0.02). The highest performance of the statistical model, defined as model with the lowest BIC, was achieved with the inclusion of variables EZ, ONL, GCL and RNFL thickness without the variables age and eccentricity.

**TABLE 3:** Univariate models and multivariable mixed effect model with smallest BIC.

	Univariate model					Multivariate model				
	Estimate	95% C I	95% C I	*p*‐value	*n* stimuli	Estimate	95% C I	95% C I	*p*‐value	*n* stimuli
MAIA versus MP‐3 (dB)	−3.437	−3.592	−3.282	<0.0001	3.600	−3.335	−3.479	−3.190	<0.0001	3.565
GCL thickness (dB/ μm)	0.027	0.021	0.034	<0.0001	3.565	0.028	0.022	0.033	<0.0001	3.565
ONL thickness (dB/μm)	0.044	0.039	0.049	<0.0001	3.565	0.031	0.025	0.037	<0.0001	3.565
EZ thickness (dB/μm)	0.176	0.148	0.203	<0.0001	3.565	0.053	0.029	0.077	<0.0001	3.565
RNFL thickness (dB/μm)	−0.045	−0.050	−0.039	<0.0001	3.565	−0.011	−0.017	−0.005	<0.0001	3.565
Eccentricity (dB/°)	−0.276	−0.308	−0.244	<0.0001	3.600	‐	‐	‐	‐	‐
Run (dB)	0.168	−0.023	0.360	0.0852	3.600	‐	‐	‐	‐	‐
Age (dB/year)	−0.053	−0.099	−0.007	0.0227	3.600	‐	‐	‐	‐	‐

*Note*: The dependent variable is sensitivity (dB).

Abbreviations: EZ, Ellipsoid zone; GCL, Ganglion cell layer; ONL, outer nuclear layer; R°, eccentricity; RNFL, retinal nerve fibre layer.

### 3.2 Correlations between eccentricity, layer thicknesses and patient age

Table 4 shows correlations between age, eccentricity and layer thicknesses. Age shows very weak correlations with EZ thickness (*r*_s_ = −0.037), ONL thickness (*r*_s_ = 0.014), GCL thickness (*r*_s_ = −0.095) and macular RNFL (*r*_s_ = 0.036) thickness. There was a strong correlation between ONL thickness and retinal eccentricity (*r*_s_ = −0.859) and RNFL thickness (*r*_s_ = −0.728), while the correlation between EZ thickness and eccentricity was moderate (*r*_s_ = −0.350).

**TABLE 4:** Spearman correlations and Multicollinearity between independent variables.

Variable	R°	Age	EZ (μm)	ONL (μm)	GCL (μm)	RNFL (μm)
R°	‐	0.001	−0.350	−0.859	−0.186	0.711
Age		‐	−0.037	0.014	−0.095	0.036
EZ (μm)			‐	0.386	0.165	−0.261
ONL (μm)				‐	0.098	−0.728
GCL (μm)					‐	−0.008
RNFL (μm)						‐

Abbreviations: EZ, Ellipsoid zone; GCL, Ganglion cell layer; ONL, outer nuclear layer; R°, eccentricity; RNFL, retinal nerve fibre layer.

## 4 DISCUSSION

PWS was significantly influenced by EZ thickness, ONL thickness and GCL RNFL thickness in a healthy ageing cohort based on a pointwise co‐registration between semi‐automatically quantified structural biomarkers on OCT and retinal sensitivity on MP. We provide precise quantitative reference values for structural biomarkers in OCT and matching functional information in healthy aged retinal tissue, as the literature on this topic remains scarce with concurrent rising relevance of functional testing through different retinal pathologies (Palkovits et al., 2018, 2021).

The EZ was automatically quantified and defined between the inner border of the EZ and the outer border of the interdigitation zone based on previous publications (Orlando et al., 2020; Riedl et al., 2022). Our study contributes to the existing literature by providing evidence that a reduction in EZ thickness in healthy aged subjects is associated with localized functional decline in retinal sensitivity, despite the absence of morphological alterations. Comparison of our overall foveal, parafoveal and perifoveal measurements with previous literature poses a challenge due to deviating measurement borders of the EZ layer (Ross et al., 2015; Srinivasan et al., 2008). Nonetheless, our EZ thickness measurements are comparable with measurements between the inner boundary of photoreceptor inner segment/outer segment junction (corresponding to inner boundary of EZ) and the inner boundary of the cone outer segment tips (corresponding to inner boundary of interdigitation zone) in other healthy subjects in the literature (Srinivasan et al., 2008). In our model EZ thickness had the highest functional impact on retinal sensitivity among structural parameters, which highlights the importance of EZ assessment. This represents a significant area of interest, as novel therapeutic targets for AMD treatment have been shown to impact EZ integrity (Birner et al., 2024; Reiter et al., 2024; Riedl et al., 2022; Schmidt‐Erfurth et al., 2024; Vogl et al., 2023).

Interestingly, in a normative database of layer thicknesses in OCT, ONL thickness was stronger correlated to increased age compared to other retinal layer thickness measurements (Palazon‐cabanes et al., 2020). We add to the literature by correlating previously reduced ONL thickness with functional decline. Also, ONL thinning was described as an important biomarker for disease progression in intermediate AMD (Aresta et al., 2024). Importantly, our results are consistent with previous studies regarding an increased thickness of RNFL towards the optic nerve and increased thickness of EZ and ONL towards the foveal centre point (Palazon‐cabanes et al., 2020; Srinivasan et al., 2008). This is in line with strong correlations between ONL and RNFL in our cohort (Table 4).

Thinning of the GCL complex was previously described in patients with non‐proliferative diabetic retinopathy (NPDRP) (van Dijk et al., 2009, 2012). This GCL thinning is caused by diabetic retinal neurodegeneration that precedes clinically detectable retinal vasculopathies and is associated with alterations in the retinal capillary network, resulting in impaired retinal sensitivity in MP (Montesano et al., 2017; van Dijk et al., 2009, 2012).

We demonstrate that thinning of GCL is associated with lower PWS in this healthy ageing cohort. This is of great interest as a reference point for future structure–function correlations in patients with early diabetic retinopathy. Interestingly, negative correlation between GCL thinning and PWS were described in individuals with systemic diabetes mellitus without retinal involvement (Montesano et al., 2017).

RNFL thinning and visual field defects are hallmarks of glaucoma (Weinreb & Tee Khaw, 2004). Preliminary use of MP in glaucoma revealed that useful additional information can be gained for, e.g., fixation stability and the determination of the preferred retinal locus (Scuderi et al., 2022). Additionally, precise structure–function correlations of the GCL are possible (Scuderi et al., 2022). Since thinning of the RNFL and losing fixation stability appear early in the disease course (Miki et al., 2014), MP may be a useful tool for early glaucoma assessment. In our cohort PWS increased with decreasing macular RNFL thickness. In studies evaluating the RNFL in glaucoma using MP, the stimuli were centred around the optic disc (Arrico et al., 2016; Kita et al., 2018). Therefore, we postulate that this counterintuitive finding was produced by our fovea‐centred grid with the highest density of MP stimulus points in the central 1 mm and physiological changes in RNFL thickness as demonstrated in Figure 2 and Figure S2. Hence, the central 1 mm physiologically attributes to higher sensitivity values, while simultaneously providing the lowest RNFL measurements. Although standard automated perimetry remains the gold standard in glaucoma diagnostics (Delgado et al., 2002; Scuderi et al., 2008), MP may provide additional information about the integrity of the macula (Kita et al., 2018; Scuderi et al., 2022).

Since the estimates of all the structural parameters were rather small, the clinical significance remains to be determined. The EZ and the ONL together representing the photoreceptors show the highest estimates among the structural parameters, emphasizing the importance of the integrity of these layers for macular retinal sensitivity. Additionally, our reference data for the inner retinal layers may enhance understanding of disease course and diagnostics of diseases like AMD, DRP and glaucoma.

Pfau et al. analysed mesopic MP sensitivity values in a multicentre setting to provide extensive evidence on variations in visual sensitivity and define contributors for visual sensitivity prediction. This large database proved increasing age (−0.4 dB) and retinal eccentricity (−0.3 dB) as factors for retinal sensitivity reduction (Pfau et al., 2024). Importantly, this work is based exclusively on functional data, while our study focuses on the correlation between morphology and function. Still, our results are in line with this large normative database with identical estimates for the reduction of PWS with increasing eccentricity (both studies −0.3 dB) (Pfau et al., 2024). Higher subject age in our study might contribute to lower estimates for age (−0.053 dB vs. −0.4 dB in Pfau et al.). Nonetheless, the predictive capacity of our model was enhanced when age was not incorporated into the final model with the highest model performance with EZ and ONL thickness. Additionally, the correlation between age and layer thickness measurements was found to be exceedingly weak (Table 4). This indicates that individual structural integrity of the retinal layers is an important contributor to retinal sensitivity in healthy aged individuals and highlights the importance of personalized structural assessment in clinical practice and for the design of clinical trials.

In 1993, Curcio et al. histologically analysed photoreceptor topography in a cohort of 27 donor eyes. Results showed a consistent decrease in the numbers of rods at the cellular level, which strongly correlated with age. No age‐related loss of cones was evident (Curcio et al., 1996). We measured significantly lower PWS in MAIA with mesopic conditions compared to MP‐3 in photopic conditions (−3 dB). This result could support the hypothesis of early rod degeneration in ageing eyes which results in lower PWS. However, the decibel outcomes are primarily dependent on the background luminosity setting, which differ between both devices. We also proved that no significant differences are present between two runs on the same device, which confirms structure–function correlations in MP as reliable outcome measurements. Precise repeatability statistics, examination time and fixation stability metrics of this cohort were previously published by our group and are in line with recent literature (Coulibaly et al., 2024).

The strengths of this study are the pointwise structure–function correlation based on 1:1 co‐registration between OCT and MP in two different MP devices. In addition, we provide a reference for retinal sensitivity values in healthy aged patients in the two most commonly used MP devices. With rising incidence in retinal disease after the age of 50 (Klein & Klein, 2013), additional references for retinal structure in healthy aged eyes is much needed. This study is subject to several limitations. Firstly, the cohort size was relatively small, with only 20 patients included. However, to prevent intraindividual correlations, we only included one eye per patient and correlated the structural changes on a point‐to‐point basis with dB and layer thickness values from 3600 MP points. Secondly, this was an exploratory analysis, hence the interpretation of the p‐values is descriptive. Importantly, as the estimates of the structural parameters remain very low, therefore, the clinical relevance of these findings remains to be determined.

In conclusion, this is the first analyses between multiple semi‐automated retinal layer measurements and functional outcomes in two different MP devices using pointwise registration between OCT and MP. This proof‐of principle study provides normative data on pointwise topographic changes of EZ, ONL and GCL layer and supports evidence on functional decline with decreased thickness of each respective layer while correcting for retinal eccentricity and subject age.

## Supporting information


Figure S1.

Figure S2.

Figure S3.

Data S1.
