# Supplementary figures and images for: Normative prospective data on automatically quantified retinal morphology correlated to retinal function in healthy ageing eyes by two microperimetry devices
Source: Acta Ophthalmol. 2024 Dec 27;103(4):423–31. doi: 10.1111/aos.17434 (PMC12069971; doi:10.1111/aos.17434)

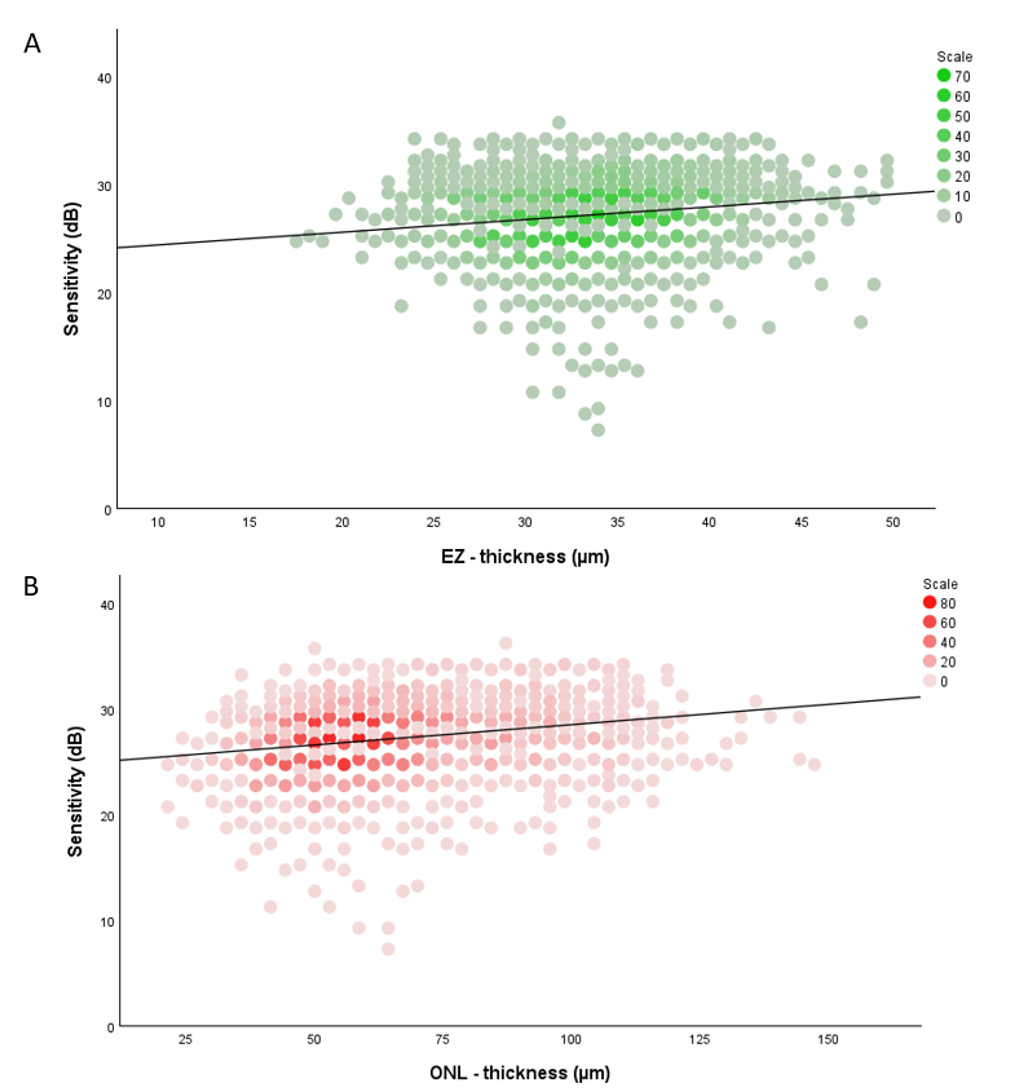

Supplement: Supplementary file 1 — Figure S1. [file AOS-103-423-s001.tif]

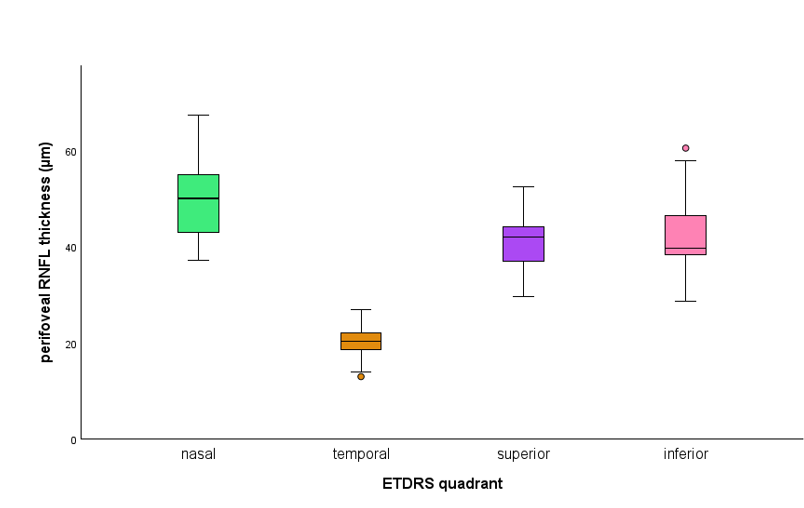

Supplement: Supplementary file 2 — Figure S2. [file AOS-103-423-s004.tif]

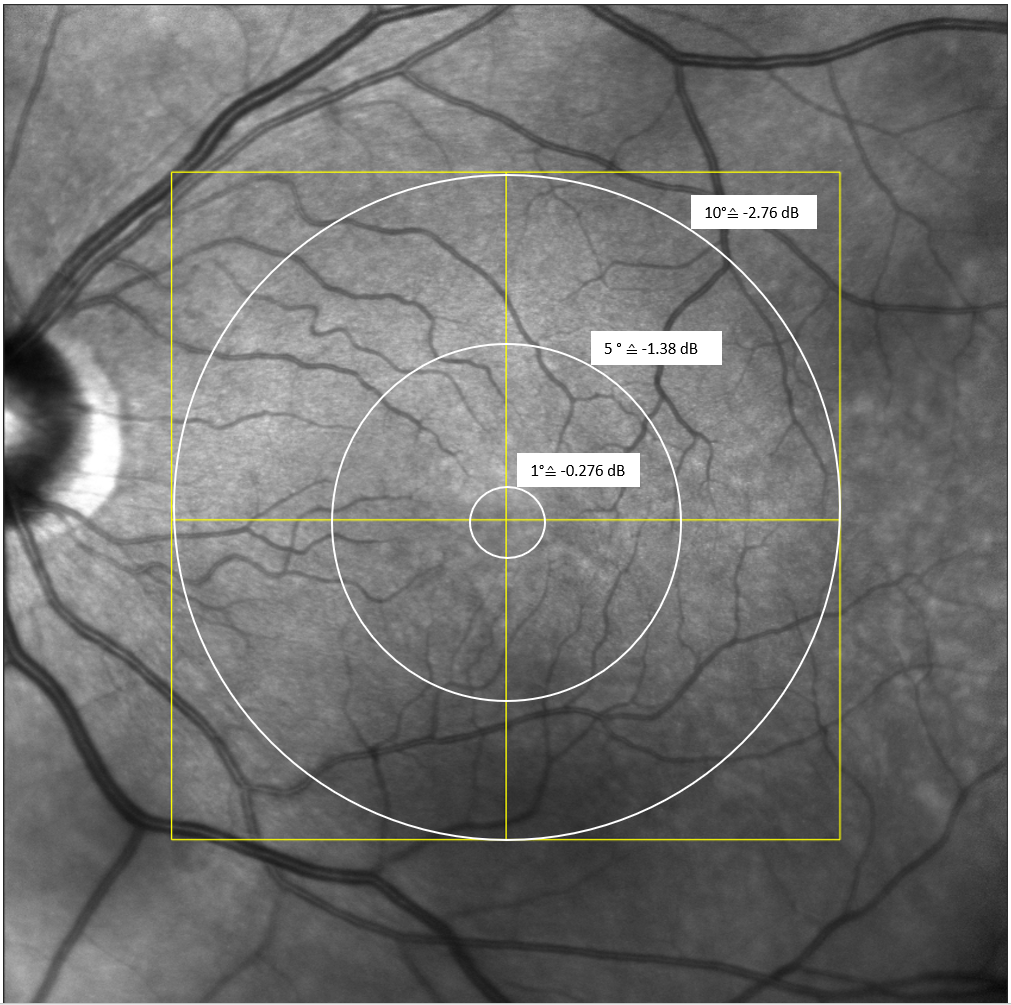

Supplement: Supplementary file 3 — Figure S3. [file AOS-103-423-s002.tif]
