# Supplementary material for: Normative prospective data on automatically quantified retinal morphology correlated to retinal function in healthy ageing eyes by two microperimetry devices
Source: Acta Ophthalmol. 2024 Dec 27;103(4):423–31. doi: 10.1111/aos.17434 (PMC12069971; doi:10.1111/aos.17434)
Supplement: Supplementary file 4 — Data S1. [file AOS-103-423-s003.docx]

**Supplemental files:**

Supplementary Table 1: Effect on eccentricity on the PWS. Note the inverse association between eccentricity and PWS.

| R° – eccentricity in degrees | Decibels (dB) |
| --- | --- |
| 0° | 0 |
| 1° | -0.276 |
| 2° | -0.552 |
| 3° | -0.828 |
| 4° | -1.104 |
| 5° | -1.380 |
| 6° | -1.656 |
| 7° | -1.932 |
| 8° | -2.208 |
| 9° | -2.484 |
| 10° | -2.760 |

**
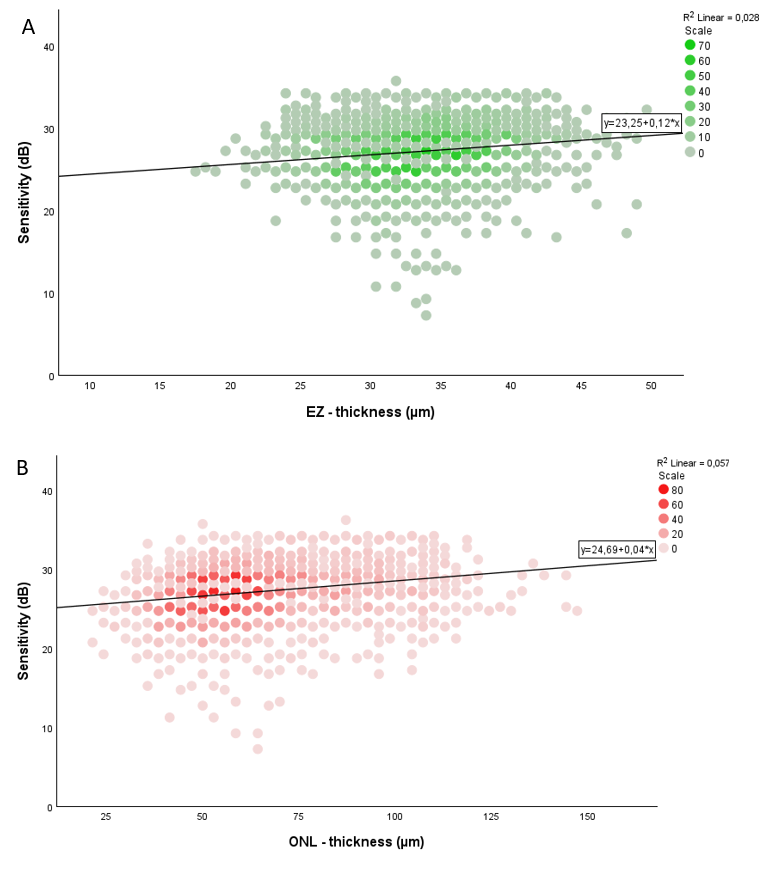
**

Supplementary Figure 1: Scatterplots showing the correlation between pointwise sensitivity (dB) and EZ thickness (A) and ONL thickness (B) measured in µm.


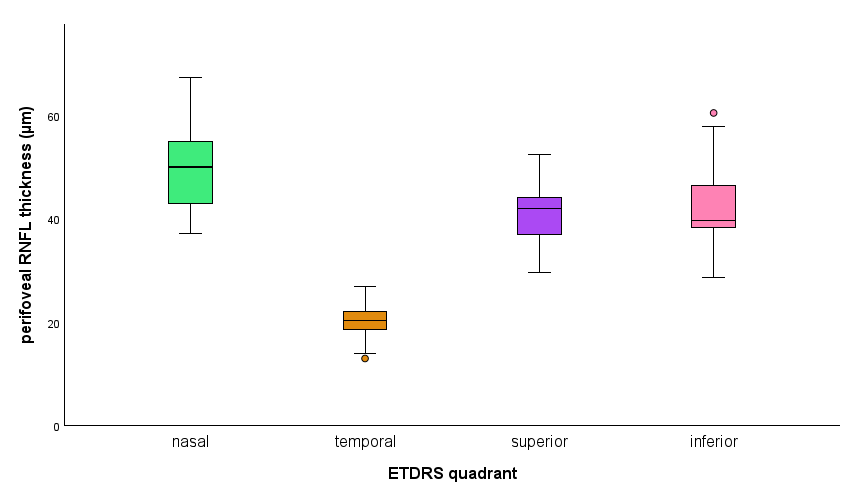


Supplementary Figure 2: RNFL thickness in μm stratified by ETDRS quadrants: Paranasal 23.75 ± 3.53, Paratemporal 19.01 ± 2.32, Parainferior 26.38 ± 2.90, Parasuperior 26.83 ± 5.04, Perinasal 50.52 ± 8.89, Peritemporal 20.00 ± 3.17, Perisuperior 41.81 ± 6.79, Periinferior 42.73 ± 8.06. Note the high and low thicknesses in the nasal and temporal quadrants, respectively. This is due to the anatomical course of the retinal nerve fibres toward the optic disc nasal to the fovea.


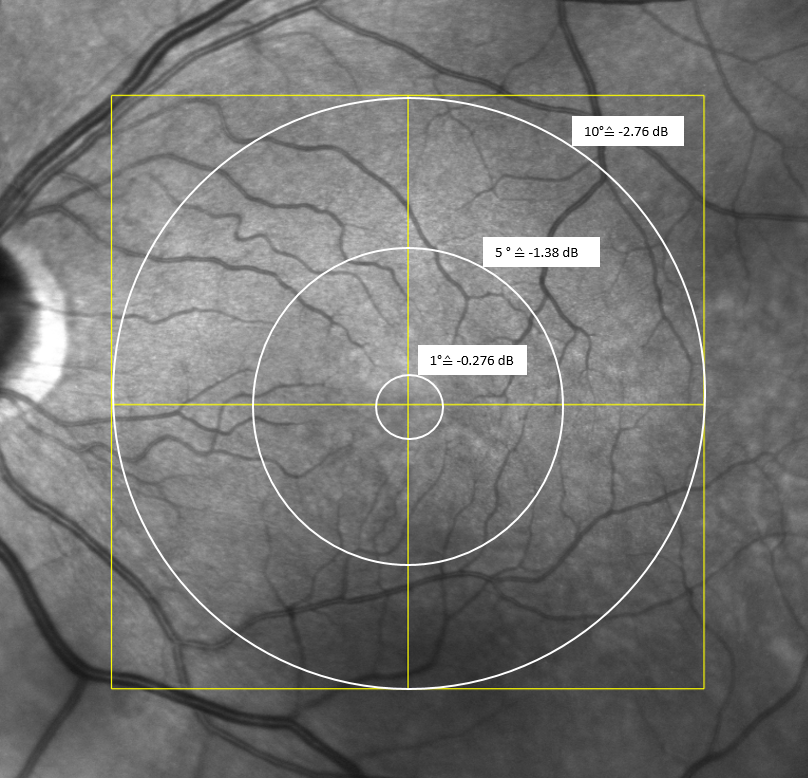


Supplementary Figure 3: Schematic representation of sensitivity loss with increasing foveal eccentricity projected onto a NIR of a left eye. The scan was acquired using a 20x20° frame marked by the yellow box. The degrees of foveal eccentricity are indicated by the white circles. With each degree of eccentricity, the PWS decreases by -0.276 dB. This results in -1.38 dB and -2.76 dB at 5° and 10°, respectively.
